# Associated Effect of *SLC40A1* and *TMPRSS6* Polymorphisms on Iron Overload

**DOI:** 10.3390/metabo12100919

**Published:** 2022-09-29

**Authors:** Lorena Duca, Francesca Granata, Elena Di Pierro, Valentina Brancaleoni, Giovanna Graziadei, Isabella Nava

**Affiliations:** Fondazione IRCCS Ca’ Granda Ospedale Maggiore Policlinico, U.O.C. Medicina Generale, 20122 Milano, Italy

**Keywords:** *SLC40A1*, *TMPRSS6*, ferroportin, matriptase-2, iron, iron overload, iron deficiency, hemoglobin, ferritin, transferrin saturation

## Abstract

Mutations in the ferroportin (FPN) gene *SLC40A1* alter iron recycling and cause disturbances in iron homeostasis. The variants of *TMPRSS6* contribute to the development of iron deficiencies. In this study, we determined the role of FPN and *TMPRSS6* gene polymorphisms in the modulation of iron homeostasis based on biochemical parameters. PCR analysis and sequencing were performed to determine the single nucleotide polymorphisms (SNPs) *SLC40A1* c.44–24G>C (rs1439816), *SLC40A1* c.663T>C (rs2304704), and *TMPRSS6* c.2207T>C (rs855791). Hemoglobin concentration and iron status were determined by standard procedures. We studied 79 iron-loaded individuals for *SLC40A1* polymorphisms. Interestingly, 35/79 individuals with *SLC40A1* SNPs also carried a *TMPRSS6* c.2207T>C polymorphism. The biochemical values of the iron overloaded individuals were compared to those of the individuals carrying *TMPRSS6* SNPs and the healthy individuals (wild-type group). The ferritin concentration, transferrin saturation % (TS%), and hemoglobin concentration were significantly higher in the participants with FPN SNPs than in the other three groups. The ferritin concentration and TS% were higher in participants with both *SLC40A1* and *TMPRSS6* SNPs than in the *TMPRSS6* and wild-type groups, while hemoglobin concentration was significantly higher than that in the *TMPRSS6* SNP group only. The participants with *TMPRSS6* SNPs had significantly lower ferritin concentration, TS%, and hemoglobin concentration than all the other groups. *SLC40A1* and *TMPRSS6* SNPs might act in the opposite direction, preventing the development of severe iron overload, and the modulation of the iron status by *TMPRSS6* SNPs might provide protection.

## 1. Introduction

Iron homeostasis is fundamental for maintaining biological functions in humans and involves the interplay between iron supply from duodenal absorption, macrophage recycling, and liver storage and iron requirements for oxygen transport, the immune system, and cognitive functions. Although iron is biologically essential, it can also be potentially toxic at high levels. Thus, it is tightly regulated at the cell and systemic levels to prevent both deficiency and overload [1]. The liver peptide hepcidin is the master regulator of systemic iron homeostasis. It controls serum iron by degrading ferroportin (FPN) in iron-absorptive enterocytes and iron-recycling macrophages [2]. Sub-optimal production of hepcidin or dysregulation of the hepcidin–FPN axis can lead to iron overload.

The active form of hepcidin functions by binding to its FPN receptor specifically expressed on the basolateral membrane of duodenal enterocytes, the membrane of splenic macrophages that recycle iron in red blood cells, and the membrane of hepatocytes. After FPN is bound by hepcidin, it is internalized and degraded; this inhibits the release of circulating iron. The expression of hepcidin is driven by changes in the levels of circulating and tissue iron.

Ferroportin is encoded by the *SLC40A1* gene that localizes on chromosome 2 (2q32) [3]. The highly conserved protein FPN, also known as IREG1 or MTP1, which plays a key role in elemental iron acquisition and transfer, was simultaneously discovered by three separate groups [4]. The protein consists of 571 amino acid residues with 12 transmembrane domains and both cytosolic N- and C-termini [5].

Mutations in the FPN gene impair iron recycling and affect iron homeostasis. Ferroportin disease, also described as hereditary hemochromatosis Type IV, HFE 4 (Online Mendelian Inheritance in Man, OMIM number 606069), unlike classical hereditary hemochromatosis, is an autosomal dominant disorder characterized by iron accumulation in macrophages of the reticuloendothelial system [6], and FPN gene knockout in the duodenum impairs iron absorption [7].

Ferroportin disease is a clinically heterogeneous iron overload syndrome. The canonical form, presenting as hepatic and spleen iron overload, is characterized by hyperferritinemia, normal to low transferrin saturation, and Kupffer cell iron storage [8]. Additionally, increased transferrin saturation, hepatocellular iron overload, hyperferritinemia, and macrophage iron loading are considered to be typical features of the non-classical phenotype [8].

1. Most patients presenting classical hemochromatosis are homozygous for the missense mutation c.845G>A (C282Y) in the HFE gene, but due to incomplete penetrance of this genotype and other environmental or genetic factors, a wide spectrum of phenotypic expression occurs for the c.845G>A homozygous condition. Diet, alcohol, gender, and viral hepatitis influence the clinical presentation of hemochromatosis [3]. Several studies were conducted to determine the role of putative genetic modifiers in iron overload and found that the ferroportin gene polymorphic variants play a key role.
2. Altès et al. [9] studied hemochromatosis patients with the classical HFE homozygous mutation and the c.44–24G>C *SLC40A1* polymorphism (rs1439816) and associated this FPN gene polymorphism with the amount of iron overload, the presence of liver disease, and, consequently, clinical aggressiveness. In another study, the presence of at least one C allele of the c.44–24G>C *SLC40A1* polymorphism was found to modulate the biochemical phenotype, specifically serum iron and transferrin saturation, in classical hemochromatosis patients [10].
3. Another study on patients from the South African population identified significant associations between c.44–24G>C and c.663T>C *SLC40A1* polymorphisms and iron overload [11]. The c.44–24G>C SNP, located in the 5′ untranslated region within 24 nucleotides upstream of the start of exon 2, affects the splicing machinery, as shown by the in silico analysis conducted using Mutation Taster (https://www.mutationtaster.org (accessed on 9 August 2022)). The c.663T>C polymorphism classified as rs2304704 (also known as V221V) does not change the amino acid sequence in the protein but alters the splice site, as determined by prediction tools (https://www.mutationtaster.org (accessed on 9 August 2022)).
4. Interestingly, the allelic frequencies of the c.44–24G>C and c.663T>C *SLC40A1* SNPs were found to be significantly higher in Italian blood donors with mild-to-moderate iron overload compared to the control group. This supported the idea that polymorphisms in the ferroportin gene are important contributors to iron storage and load in apparently healthy subjects [12].

Several studies on disorders of iron metabolism indicated the existence of a genetic contribution to the development of iron deficiency [13]. Specifically, the Trans-Membrane Protease Serine 6 (*TMPRSS6*, gene locus c. 22q12.3) that encodes matriptase-2 expressed by the liver influences iron metabolism in humans and other animals.

*TMPRSS6* is an essential regulator of iron homeostasis, as it represents a physiological suppressor of hepcidin. *TMPRSS6* mutations cause iron refractory iron deficiency anemia (IRIDA) (OMIM number 206200) due to an increase in the concentrations of hepcidin, which degrades intestinal ferroportin and prevents normal iron absorption [14]. Familial and sporadic cases of IRIDA have been described in individuals of different ages with different types of *TMPRSS6* mutations and different clinical presentations. These may range from severe anemia with a parenteral iron requirement in infancy to isolated microcytosis and low transferrin saturation with unremarkable anemia in adulthood [15,16].

The expression of matriptase-2 can be modulated by the iron status [17]. In rats under acute iron deprivation, hepatic matriptase-2 protein levels are upregulated to repress hepcidin production [18]. Some studies have also found that the expression of the *TMPRSS6* mRNA is suppressed by inflammation [19] and upregulated by hypoxia [20,21] and erythropoietin [22].

*TMPRSS6* polymorphisms are associated with the risk of developing iron deficiency anemia (IDA) in individuals of European and Asian ancestry [23]. One of the principal *TMPRSS6* polymorphisms analyzed was c.2207T>C (rs855791) in exon 17. This polymorphism is responsible for a missense variant that substitutes the amino acid 736 valine with alanine (V736A), and it was first reported by Chambers et al. [24]. Its allelic frequency varies between 34% and 63%, depending on the studied population [25]. The c.2207T>C polymorphism was shown to be associated with lower hemoglobin levels, mean corpuscular volume (MCV), and serum iron in genome-wide association studies on general adult populations [26,27]. Additionally, in other concomitant pathologies, such as thalassemia, this polymorphism negatively influences the capability of iron store repletion, the degree of anemia, and the response to oral iron supplementation. Finally, the polymorphism was hypothesized to be a potential risk factor in conditions of iron malabsorption (i.e., celiac disease) [28] or iron loss in fertile women [29].

The aim of this study was to elucidate the role of polymorphisms in genes that encode FPN and matriptase-2 in the modulation of iron balance in the body by observing biochemical parameters in individuals with mild-to-severe iron overload, not due to genetic hemochromatosis, metabolic diseases, transfusion-dependent pathologies, or xenobiotic factors.

## 2. Materials and Methods

### 2.1. Study Population

Peripheral blood samples were collected from 161 individuals (mean age 44 ± 16 years). The study was conducted following the Word Medical Association’s Declaration of Helsinki for Medical Research. All participants signed a form providing informed consent for the diagnosis and research approved by the ethics committee of Fondazione IRCCS Ca’ Granda Ospedale Maggiore Policlinico of Milan (Italy). The identity of the participants was not revealed.

### 2.2. Collection of DNA Samples

Genomic DNA was extracted from the lymphomonocytes of peripheral blood samples using the Maxwell^®^ 16 SEV Blood DNA Purification Kit (Catalog No. AS1010 Promega, Madison, WI, USA) following the manufacturer’s instructions. The DNA samples were stored at −20 °C until use.

### 2.3. Genotyping for Common Polymorphisms in Ferroportin and Matriptase-2 Genes

Classical PCR and sequencing techniques were used to analyze the entire coding regions of *SLC40A1* and *TMPRSS6*. The primers used for conducting PCR and cycle sequencing of the exons containing the studied SNPs are listed in Table 1. Direct sequencing was performed using the BigDye Terminator Cycle Sequencing Ready Reaction Kit v3.1 for AbiPrism 310 Genetic Analyzer (Applied Biosystems, Life Technologies Corporation, Carlsbad, CA, USA). The tested polymorphisms included *SLC40A1* c.44–24G>C (rs1439816), *SLC40A1* c.663T>C (rs2304704), and *TMPRSS6* c.2207T>C (rs855791).

### 2.4. Biochemical Analyses

Full blood panel, hemoglobin levels, and iron status (serum iron, transferrin, TS%, and ferritin) were analyzed following the standard diagnostic laboratory procedures of Fondazione IRCCS Ca’ Granda Ospedale Maggiore Policlinico.

### 2.5. Statistical Analysis

To determine the differences between the groups, a one-way ANOVA test was performed. The differences were considered to be statistically significant at *p* < 0.05. All analyses were conducted using GraphPad Prism.

Fisher’s exact test was conducted to verify the association between the simultaneous presence of *SLC40A1* and *TMPRSS6* SNPs and the serum ferritin concentration below 200 μg/L in this iron overload cohort.

A sample size of 12 was calculated to provide 95% power to the study (α error of 0.01).

## 3. Results

### 3.1. Patients

The patients (48 ± 17 years, n = 79) who were diagnosed with iron overload were examined for *SLC40A1* c.44–24G>C (rs1439816) and c.663T>C (rs2304704) polymorphisms after they were excluded as carriers of genetic hemochromatosis, metabolic diseases, transfusion-dependent pathologies, other possible causes of secondary iron overload, liver disease, and inflammation. None of them had unusual dietary habits or consumed abnormal levels of ethanol that could explain their iron overload. As some individuals showed borderline values for ferritin or TS% even though they were not subdued to phlebotomy or chelation therapy, we investigated all subjects for the *TMPRSS6* c.2207T>C SNP (rs855791), which is associated with the risk for the development of IDA [23].

### 3.2. Genetic Analysis

Of the 79 patients presenting iron overload, 44 had *SLC40A1* polymorphisms only, 19 showed one of the two polymorphisms in homozygosis or heterozygosis, and 25 showed both polymorphisms. Additionally, 35 individuals with *SLC40A1* polymorphisms also carried the *TMPRSS6* c.2207T>C SNP (Table 2 and Table 3).

For every SNP studied, the allelic frequencies were analyzed, and the obtained data were compared to the allelic frequencies obtained from the most important projects on common human genetic variations (Table 4). The allelic frequencies of *SLC40A1* SNPs in our study were similar to those of the 1000 Genomes, ALFA, and TOPMED projects. The allelic frequency of *TMPRSS6* c. 2207T>C in our study was lower than that reported in the resources on human genetic variation, as expected in a selected population for iron overload.

### 3.3. Biochemical Analysis

The biochemical parameters of all 79 participants were evaluated, and the results were as follows: ferritin 443 ± 47 μg/L, TS% 34.0 ± 2.0%, and hemoglobin 13.2 ± 0.2 g/dL (values are expressed as the mean ± standard error).

These participants were then divided into two groups based on their genotypes. The first group only consisted of *SLC40A1* SNP carriers, while the second group consisted of individuals with both *SLC40A1* and *TMPRSS6* polymorphisms.

The obtained data were compared to the biochemical parameters of a previously studied group of 47 patients who lacked variants in the *SLC40A1* gene and carried the *TMPRSS6* c.2207T>C SNP, 22 of whom exhibited homozygosis, and 25 exhibited heterozygosis. These patients were referred to the Rare Disease Centre of Fondazione IRCCS Ca’ Granda Ospedale Maggiore Policlinico of Milan (Italy) for persistent IDA without celiac disease, gastrointestinal bleeding, or *Helicobacter pylori* infection.

Additionally, 35 healthy participants with wild-type genotypes for the two investigated genes were selected, and they were matched for sex and age and included in the control group for total blood count and iron parameters.

The ferritin concentration, TS%, and hemoglobin concentrations were significantly higher in the participants carrying only FPN gene polymorphisms than in the other three groups. The ferritin concentration and TS% were significantly higher in the participants with both *SLC40A1* and *TMPRSS6* SNPs than in the participants with *TMPRSS6* SNPs and the healthy participants (wild-type); furthermore, the hemoglobin concentration was significantly higher than that in the participants with the *TMPRSS6* polymorphism only. The ferritin concentration, TS%, and hemoglobin concentration were significantly lower in the participants with *TMPRSS6* SNPs only than in the participants of all other groups (all *p*-values are reported in Table 5 and its caption).

The results of Fisher’s exact test showed that the simultaneous presence of *SLC40A1* and *TMPRSS6* SNPs was strongly associated with serum ferritin concentration below 200 μg/L in this iron overload cohort (*p* < 0.00001; α error of 0.01).

## 4. Discussion

The regulation of ferroportin expression is complex, with strictly controlled mechanisms at the transcriptional, post-transcriptional, and post-translational levels. This indicates that the control of systemic iron flux under different conditions is flexible [30]. In this study, the high variability of the FPN gene was observed in a group of 79 patients without HFE mutations or secondary causes of iron overload but presenting alterations in TS% and serum ferritin levels. Specifically, among 44 carriers of *SLC40A1* SNPs only, 19 individuals showed *SLC40A1* c.44–24G>C or c.663T>C SPNs in homozygosis or heterozygosis, and 25 showed both polymorphisms. Their iron overload parameters were significantly higher than those in the control group of healthy individuals (wild-type) for the studied genes. These observations were consistent with the findings of previous studies, where it was shown that the polymorphic variants of *SLC40A1* c.44–24G>C (rs1439816) and c.663T>C (rs2304704) had a significant effect on iron metabolism and could explain the considerable unrelated HFE phenotypic variability that might exist in iron overload [9,12]. Moreover, 35 of the 79 individuals carried *SLC40A1* SNPs and the *TMPRSS6* c.2207T>C polymorphism together. Ferritin concentrations and TS% were significantly lower in these individuals than in the carriers of *SLC40A1* SNPs only and higher than in the individuals carrying the *TMPRSS6* SNP only and the healthy individuals (wild-type). Additionally, the iron and hemoglobin parameters were significantly lower in participants carrying the *TMPRSS6* SNPs only than in the individuals carrying the *SLC40A1* SNPs, the *SLC40A1* and *TMPRSS6* SNPs, and the healthy participants (wild-type). These results were similar to those of genome-wide association studies, where it was shown that the *TMPRSS6* c.2207T>C polymorphism is associated with lower transferrin saturation, hemoglobin concentration, and MCV levels in the general adult population [26,31]. Based on the findings in this study, we detected an intermediate iron status phenotype in individuals carrying both *SLC40A1* polymorphisms and *TMPRSS6* c.2207T>C SNP relative to the participants carrying either *SLC40A1* or *TMPRSS6* SNP only. *TMPRSS6*, which codes for matriptase-2, and *SLC40A1*, which codes for ferroportin, are upstream and downstream of hepcidin production, respectively. Hence, alterations in the function of matriptase-2 might lead to insufficient downregulation of hepcidin expression and increased internalization of ferroportin. On the other hand, modifications of the ferroportin protein prevent its internalization and destruction by hepcidin, causing iron overload. Therefore, based on the mechanism of action and regulation of hepcidin and the presence of an intermediate iron status phenotype in individuals carrying both *SLC40A1* and *TMPRSS6* SNPs, our results support the hypothesis that *SLC40A1* and *TMPRSS6* SNPs modulate iron homeostasis by acting in the opposite direction and preventing a severe iron overload. Moreover, the *TMPRSS6* c.2207T>C SNPs might protect individuals carrying variants in genes responsible for iron overload by modulating iron homeostasis.

## Figures and Tables

**Table 1:** The list of primer sequences and annealing temperatures for PCR and sequencing used for detecting the studied polymorphisms.

*Gene* Polymorphism	Primer Sequences (Forward and Reverse)	Amplicon Length (bp)	Annealing Temperature (°C)
*SLC40A1* c.44–24G>C (rs1439816)	5′-GTGGGCAGAGCAGGAGAGAAG-3′ 5′-GATGTGAGCAAAGGGCCAGAC-3′	371	61
*SLC40A1* c.663T>C (rs2304704)	5′-AACGAAATACATCGGTTCATAGG-3′ 5′-ATTAAAGCATGTGTACTTGGATG-3′	495	58
*TMPRSS6* c.2207T>C (rs855791)	5′-GGAATCTATACTCTTGGTTTACAG-3′ 5′-CTTGCCTCGTCTACCAAAGCG-3′	337	61

**Table 2:** The number and genotype percentage of participants carrying *SLC40A01* only. The genotype percentage is given in brackets.

Genotype of Subjects Carrying Only *SLC40A1* Polymorphism: 44 (100)	
c.[44–24G>C];[44–24G>C]	3 (6.8)
c.[44–24G>C];[=]	2 (4.5)
c.[663T>C];[663T>C]	7 (16)
c.[663T>C];[=]	7 (16)
c.[44–24G>C];[44–24G>C] c.[663T>C];[663T>C]	9 (20.4)
c.[44–24G>C];[44–24G>C] c.[663T>C];[=]	14 (32)
c.[44–24G>C];[=] c.[663T>C];[663T>C]	0 (0)
c.[44–24G>C];[=] c.[663T>C];[=]	2 (4.5)

**Table 3:** The number and genotype percentage of participants carrying both *SLC40A01* and *TMPRSS6* SNPs. The genotype percentage is given in brackets.

Genotype of Subjects Carrying Both *SLC40A1* and *TMPRSS6* Polymorphisms: 35 (100)		
	*TMPRSS6*	
	c.[2207T>C];[2207T>C]	c.[2207T>C];[=]
***SLC40A1***		
c.[44–24G>C];[44–24G>C]	1 (2.9)	2 (5.7)
c.[44–24G>C];[=]	0 (0)	1 (2.9)
c.[663T>C];[663T>C]	0 (0)	1 (2.9)
c.[663T>C];[=]	0 (0)	0 (0)
c.[44–24G>C];[44–24G>C] c.[663T>C];[663T>C]	4 (11.2)	5 (14.3)
c.[44–24G>C];[44–24G>C] c.[663T>C];[=]	7 (20.0)	9 (25.7)
c.[44–24G>C];[=] c.[663T>C];[663T>C]	0 (0)	1 (2.9)
c.[44–24G>C];[=] c.[663T>C];[=]	3 (8.6)	1 (2.9)

**Table 4:** The allelic frequencies of the analyzed SNPs in the studied population compared to those in the 1000 Genomes, ALFA, and TOPMED projects.

Polymorphisms	Study Population	1000 Genomes Study Global Population	ALFA Project Total Population	TOPMED Program
*SLC40A1* c.44–24G>C rs1439816	G 0.74683	G = 0.6595	G = 0.78005	G = 0.682670
	C 0.25317	C = 0.3405	C = 0.21995	C = 0.317330
*SLC40A1* c.663T>C rs2304704	T 0.38608	T = 0.4499	T = 0.392810	T = 0.477706
	C 0.61392	C = 0.5501	C = 0.607190	C = 0.522294
*TMPRSS6* c.2207T>C rs855791	T 0.6835	T = 0.3954	T = 0.439967	T = 0.361479
	C 0.3165	C = 0.6046	C = 0.560033	C = 0.638521

**Table 5:** Biochemical parameters of the individuals carrying *SLC40A1* (44–24G>C and/or 663T>C) polymorphisms only, indicated as *SLC40A1*, both *SLC40A1* and *TMPRSS6* c.2207T>C SNPs (*SLC40A1* and *TMPRSS6*), and only *TMPRSS6* c.2207T>C polymorphism (*TMPRSS6*), compared to the wild-type healthy group of participants (Wild-type).

Polymorphisms	Number of Subjects	Serum Ferritin (μg/L)	Transferrin Saturation %	Hemoglobin (g/dL)
*SLC40A1*	44	702 ± 57 *	40.2 ± 2.2 ^	13.5 ± 0.2 •#°
*SLC40A1* and *TMPRSS6*	35	108 ± 23 °#	25.7 ± 2.9 °#	12.8 ± 0.4 °
*TMPRSS6*	47	8 ± 1 √	7.1 ± 0.7 √	10.5 ± 0.3 ∞
Wild-type	35	49 ± 7	19.6 ± 1.4	12.5 ± 0.2

Values are reported as mean ± standard error. *: *p* < 0.0001 versus *SLC40A1* and *TMPRSS6*, *TMPRSS6* and wild-type groups; °: *p* < 0.001 versus *TMPRSS6* group; #: *p* = 0.02 versus wild-type; ^: *p* < 0.001 versus *SLC40A1* and *TMPRSS6*, *TMPRSS6* and wild-type groups; •: *p* = 0.04 versus *SLC40A1* and *TMPRSS6*; √: *p* < 0.0001 versus *SLC40A1*, *SLC40A1* and *TMPRSS6*, and wild-type groups; ∞: *p*< 0.001 versus *SLC40A1*, *SLC40A1* and *TMPRSS6*, and wild-type groups.

## Data Availability

The data presented in this study are available on request from the corresponding author.
